# A High Percentage of Patients Recovered From COVID-19 but Discharged With Abnormal Liver Function Tests

**DOI:** 10.3389/fphys.2021.642922

**Published:** 2021-03-17

**Authors:** Qinyi Gan, Beilei Gong, Manli Sun, Zhujun Cao, Yuyan Zheng, Yajie Zhang, Pengfei Wen, Yuanbing Shen, Lei Hong, Tingting Hou, Yuqin Jia, Wei Li, Hecheng Li, Qing Xie

**Affiliations:** ^1^Department of Infectious Diseases, Ruijin Hospital, Shanghai Jiao Tong University School of Medicine, Shanghai, China; ^2^The First Affiliated Hospital of Bengbu Medical College, Bengbu, China; ^3^Clinical Research Center for Respiratory Disease in Anhui Province, Bengbu, China; ^4^Department of Medicine and Education, Bengbu Infectious Disease Hospital, Bengbu, China; ^5^Department of Thoracic Surgery, Ruijin Hospital, Shanghai Jiao Tong University School of Medicine, Shanghai, China; ^6^Bengbu Institute of Medical Sciences, Bengbu, China; ^7^Bengbu City Center for Disease Control and Prevention, Bengbu, China

**Keywords:** SARS-CoV-2, COVID-19, coronavirus, liver function tests, risk factor

## Abstract

**Background:**

Coronavirus disease 2019 (COVID-19) pandemic has become the most severe global health issue. Abnormal liver functions are frequently reported in these patients. However, liver function abnormality was often overlooked during COVID-19 treatment, and data regarding liver functions after cure of COVID-19 is limited. This study aimed to reveal the changes of liver function tests (LFTs) during hospitalization, and its clinical significance in patients with COVID-19.

**Methods:**

In this retrospective, bi-center study, a total of 158 hospitalized patients diagnosed with COVID-19 in China were included from January 22nd, 2020 to February 20th, 2020. Clinical features, laboratory parameters including LFTs, and treatment data were collected and analyzed. LFTs included alanine transaminase, aspartate aminotransferase, alkaline phosphatase, gamma-glutamyl transferase, and total bilirubin. Patients were considered with abnormal LFTs when any value of these tests was higher than upper limit of normal.

**Results:**

Of 158 patients with COVID-19, 67 (42.41%) patients had abnormal LFTs on admission and another 50 (31.65%) patients developed abnormal LFTs during hospitalization. The incidence of LFTs abnormality in severe COVID-19 cases was significantly higher than non-severe cases. All LFTs in COVID-19 patients were correlated with oxygenation index. There was no statistical difference in treatment between the patients with or without liver test abnormalities. By the time of discharge, there were still 64 (40.50%) patients with abnormal LFTs. Logistic regression analysis identified younger age, hypertension and low lymphocyte counts as independent risk factors for persistent abnormal LFTs during hospitalization.

**Conclusion:**

Liver function tests abnormality was common in COVID-19 patients and was more prevalent in severe cases than in non-severe cases. A substantial percentage of patients still had abnormal LFTs by the time of discharge.

## Introduction

The coronavirus disease 2019 (COVID-19), caused by severe acute respiratory syndrome coronavirus 2 (SARS-CoV-2), has posed a serious threat to the global public health (Zhu et al., 2020). The main manifestations of COVID-19 include fever, dry cough, fatigue, vomit, and respiratory distress. Despite atypical pneumonia as the primary symptom, liver impairment has been observed in many clinical cases (Wang et al., 2020; Chen et al., 2020a). In patients with SARS-CoV infection, liver dysfunction has also been reported as a common clinical manifestation (Humar et al., 2004), but is most often mild. Previous studies suggested that patients with abnormal liver tests had significantly higher odds of developing severe pneumonia, and medication such as lopinavir/ritonavir may increase the risks of liver injury (Bloom et al., 2020; Cai et al., 2020).Hence, a surveillance of liver function change and outcome of COVID-19 patients is necessary. However, the clinical significance, underlying mechanisms and changes of liver function abnormality during hospitalization and its risk factors in COVID-19 patients are still unclear.

The current study aimed to investigate the clinical characteristics and changes of liver function tests (LFTs) during hospitalization in patients with COVID-19.

## Methods

### Study Design

This was a retrospective study performed in two government-designated COVID-19 treatment units from Anhui province, China (the Bengbu Infectious Disease Hospital and the First Affiliated Hospital of Bengbu Medical College). The study protocol was reviewed and approved by the Ethics Committee of Bengbu Infectious Disease Hospital and the First Affiliated Hospital of Bengbu Medical College. Patient informed consent was waived by the Committee considering the retrospective design.

### Patients

Chart review was performed in all patients who admitted to the two hospitals from January 22, to February 20, 2020. Inclusion criteria were as following: in-patients with positive SARS-CoV-2 as confirmed by the RT-PCR test of the nasopharynx swab samples. Exclusion criteria: (1) age below 18 years old; (2) with established chronic liver disease; and (3) with cancer or other end-stage disease status. Laboratory tests were performed to rule out chronic liver disease, such as viral hepatitis. Abdominal ultrasound was performed on the basis of laboratory tests and patients reported medical history, if necessary.

### Main Variables

Demographic and clinical data were collected as follows: age, gender, comorbidity, symptoms, the first recorded vital signs on admission, laboratory findings on admission, and medications before and during hospitalization and length of stay. In addition to admission value, peak and value at discharge for liver biochemistry parameters were also collected, including alanine transaminase (ALT), aspartate aminotransferase (AST), alkaline phosphatase (ALP), gamma-glutamyl transferase (GGT), and total bilirubin (TB). The ratio of the partial pressure of arterial oxygen to the fraction of inspired oxygen (PaO2: FiO2) at admission was estimated for each patient with the use of methods developed by Brown and colleagues (Rice et al., 2007; Brown et al., 2017). Clinical outcome of each patient was classified into death and alive according to the vital status at discharge.

### Definition

Severity of COVID-19 was defined according to the sixth edition of the Chinese national guideline on the diagnosis and treatment of COVID-19. Patients were defined as severe COVID-19 with the presence of any of the following conditions: (1) respiration rate ≥30 times/min; (2) oxygen saturation (resting state) ≤93%; (3) PaO2: FiO2 ≤ 300 mmHg (1 mm Hg = 0.133 kPa); or (4) the occurrence of respiratory or other organ failure that requires intensive care unit (ICU) monitoring and treatment, or shock. Otherwise, patients were diagnosed as non-severe COVID-19. LFTs, including ALT, AST, GGT, ALP, and TB, was considered abnormal when any of these markers was higher than upper normal limit.

### Statistical Analysis

Categorical variables were described as count (percentages). Continuous variables with normal distribution were presented as mean ± standard deviation, otherwise as median (Inter Quartile Range). Means for continuous variables were compared using independent group *t*-tests when the data were normally distributed; otherwise, the Mann–Whitney test was used. Categorical variables were compared using the χ^2^ test or the Fisher’s exact test as appropriate. Clinical characteristics and baseline laboratory findings were compared between patients with and without liver test abnormality. Pearson’s correlation analysis was performed to determine the association of calculated PaO2/FiO2 with ALT, AST, GGT, ALP, or TB. Univariate logistic regression was performed to identify the risk factors of persistently abnormal LFTs during hospitalization, followed by multivariate logistic regression to adjust potential confounding effects. All statistical analyses were performed using Graphpad Prism 7.0 or STATA 15.1. A 2-sided α of less than 0.05 was considered statistically significant.

## Results

### Clinical Characteristics and Laboratory Results of Patients With COVID-19 on Admission

A total of 160 patients with COVID-19 were identified. Of them, 1 patient under 18 years of age and 1 patient with chronic Hepatitis B were excluded. 158 were finally included for analysis. The median age was 53 (IQR 44.5–60) years, and 76 (48.1%) were male. The most common symptoms were fever (97.5%) followed by fatigue (45.6%), cough (41.8%), and chest distress (32.9%). Among all the patients, 65 (41.1%) had one or more coexisting chronic medical conditions, of which 48 (30.4%) had hypertension and 23 (14.6%) had diabetes mellitus. A total of 121 (76.6%) patients were non-severe COVID-19 and 37 (23.4%) were severe. Laboratory findings at admission were enumerated in Table 1. Of note, 22 and 44 patients were receiving antiviral and antibiotics before admission, respectively. 5 (3.16%) patients died during hospitalization because of COVID-19 induced respiratory failure or multiple organ dysfunction syndrome.

**TABLE 1:** Baseline characteristics of patients with COVID-19.

	**Overall**	**Normal liver function**	**Abnormal liver function**	***P* value**
	**(*n* = 158)**	**(*n* = 91)**	**(*n* = 67)**	
Age, years	53 (44.5–60)	53 (43–60)	53 (45–60)	0.9308
Male gender, *n* (%)	76 (48.10%)	35 (38.46%)	40 (59.70%)	0.01
Chronic medical illness, *n* (%)	65 (41.14%)	32 (35.16%)	33 (49.25%)	0.0753
Hypertension	48 (30.37%)	24 (26.37%)	24 (35.82%)	0.2019
Diabetes mellitus	23 (14.56%)	12 (13.19%)	11 (16.41%)	0.5693
Calculated PaO2/FiO2	457.1 (442.9–461.9)	457.1 (452.4–466.7)	452.4 (442.9–461.9)	0.0121
**Disease severity status, *n* (%)**				
Severe	37 (23.42%)	17 (18.68%)	20 (29.85%)	0.1013
Non-severe	121 (76.58%)	74 (81.32%)	47 (70.15%)	
**Clinical manifestation, *n* (%)**				
Fever	154 (97.47%)	89 (97.80%)	65 (97.01%)	>0.9999
Cough	66 (41.77%)	38 (41.76%)	28 (41.79%)	0.9967
Expectoration	62 (39.24%)	34 (37.36%)	28 (41.79%)	0.5732
Fatigue	72 (45.57%)	45 (49.45%)	27 (40.30%)	0.2536
Chest distress	52 (32.91%)	25 (5.48%)	27 (40.30%)	0.09
Nausea	15 (9.49%)	10 (1.10%)	5 (7.46%)	0.4549
Diarrhea	20 (12.66%)	10 (1.10%)	10 (14.92%)	0.4621
**Laboratory result**				
Leukocyte, ×10^9^/L	5.30 (4.16–6.89)	5.24 (4.06–6.76)	5.4 (4.42–7.69)	0.1335
Neutrophil, ×10^9^/L	3.48 (2.4–4.5)	3.25 (2.12–4.23)	3.74 (2.65–5.7)	0.0137
Lymphocyte, ×10^9^/L	1.35 (0.93–1.71)	1.43 (1–1.78)	1.15 (0.84–1.67)	0.0429
Platelate, ×10^9^/L	171 (128.3–220.5)	176 (131–220)	156 (115–230)	0.3454
Hemoglobin, g/L	127.5 (117–140)	126 (118–138)	129 (116–144)	0.6936
Blood glucose, mmol/L	5.75 (5.05–6.94)	5.63 (4.95–6.91)	5.99 (5.25–7.023)	0.0907
Albumin, g/L	38.05 ± 4.57	38.12 ± 4.17	37.95 ± 5.096	0.8156
ALT, IU/L	20.8 (13–36.43)	14 (11.6–20.9)	36.9 (27.9–55.1)	<0.0001
AST, IU/L	26.1 (19.68–40.08)	20 (18–25)	41.9 (32–57.3)	<0.0001
TBIL, μmol/L	8.95 (6.58–13.03)	8.1 (6.1–11.1)	9.72 (7.19–16.1)	0.003
ALP, IU/L	54 (43–69)	50.42 ± 16.68	62 (51.75–78.25)	<0.0001
GGT, IU/L	24 (16–48.5)	18 (13–25)	57 (28.75–89.75)	<0.0001
Lactic dehydrogenase, U/L	245 (207–316; *n* = 149)	226.5 (188.3–271; *n* = 84)	291 (227.8–382.8; *n* = 65)	<0.0001
Creatine, μmol/L	61.5 (50–73)	61.48 ± 13.93	64 (49–75)	0.5224
blood urea nitrogen, mmol/L	3.7 (3.1–4.69)	3.6 (3.1–4.55)	3.82 (3.1–4.99)	0.1055
C-reactive protein, μg/dL	26.05 (7.98–72.96)	17.6 (6.17–47.73)	40.8 (17.84–102.1)	0.0007
D-dimer, μg/mL	0.55 (0.33–0.84; *n* = 106)	0.49 (0.3–0.81; *n* = 57)	0.61 (0.38–0.98; *n* = 49)	0.1235
INR	1.04 (1–1.13; *n* = 104)	1.02 (0.99–1.11; *n* = 60)	1.065 (1–1.13; *n* = 44)	0.3171
PT, s	11.9 (11.2–12.5; *n* = 104)	11.9 (11.13–12.48; *n* = 60)	12 (11.4–12.58; *n* = 44)	0.7123
**Medication before admission, *n* (%)**				
Antivirals	22 (13.92%)	16 (17.58%)	6 (8.96%)	0.1216
Antibiotics	44 (27.85%)	30 (32.97%)	14 (20.90%)	0.0943
Herbals	28 (17.72%)	17 (18.68%)	11 (16.41%)	0.7127
NSAIDs	12 (7.59%)	10 (10.99%)	2 (2.99%)	0.073

*ALT, alanine aminotransferase; AST, aspartate transaminase; ALP, alkaline phosphatase; GGT, gamma-glutamyltransferase; TBIL, total bilirubin abnormal; PT, prothrombin time; and NSAIDs, non-steroidal anti-inflammatory drugs.*

All these 158 patients were classified into patients with (*N* = 67) and without (*N* = 91) abnormal LFTs based on variables at admission. In patients with abnormal LFTs, 30 (33.0%), 45 (49.5%), 8 (8.8%), 4 (4.4%), or 40 (44.0%) patients had elevated ALT, AST, TB, ALP, or GGT, respectively. Besides, they were more likely to be male, with lower calculated PaO2/FiO2 and lymphocyte count, higher lactic dehydrogenase and C-reactive protein (Table 1).

### Association of LFTs Abnormality With Disease Severity, PaO2: FiO2 and Medications

**FIGURE 1:**
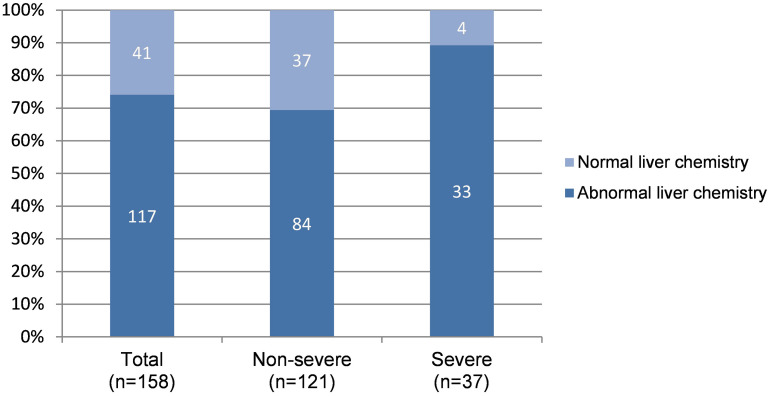
Distribution of normal and abnormal LFTs* according to severity of COVID-19. LFTs, Liver function tests. *Patients with any value of ALT/AST/ALP/GGT/TB higher than upper limit of normal range during hospitalization was considered with abnormal LFTs, otherwise with normal LFTs.

During hospitalization, additional 50 patients had abnormal LFTs, adding to 117 (74.05%) patients with abnormal LFTs. The proportion of patients with abnormal LFTs was significantly higher in severe group than in non-severe group (89.2% vs 69.4%, *p* = 0.018; Figure 1). Moreover, the value of calculated PaO2/FiO2 was negatively correlated with ALT (*r* = −0.44, *p* < 0.0001), AST (*r* = −0.43, *p* < 0.0001), TB (*r* = −0.33, *p* < 0.0001), ALP (*r* = −0.3, *p* = 0.0001), and GGT (*r* = −0.28, *p* = 0.0003; Table 2). There was no difference in the percentages of patients with abnormal LFTs between patients with and without antivirals, antibiotics, NSAIDs, or herbals (Table 3).

**TABLE 2:** Pearson’s correlation coefficients between calculated PaO2/FiO2 and LFTs.

	**Calculated PaO2/FiO2**	
	***r***	***P* value**
ALT	−0.44	<0.0001
AST	−0.43	<0.0001
TBIL	−0.33	<0.0001
AKP	−0.3	0.0001
GGT	−0.28	0.0003

*LFTs, Liver function tests; ALT, alanine aminotransferase; AST, aspartate transaminase; ALP, alkaline phosphatase; GGT, gamma-glutamyltransferase; and TBIL, total bilirubin abnormal.*

**TABLE 3:** Treatments in patients with COVID-19 according to the presence or absence of abnormal LFTs during entire hospitalization.

	**Without abnormal LFTs**	**With abnormal LFTs**	***P* value**
	**(*n* = 41)**	**(*n* = 117)**	
**Antiviral agents**			
Yes	40	116	0.4529
No	1	1	
**Antibiotics**			
Yes	23	71	0.6067
No	18	46	
**Herbal**			
Yes	33	95	0.9207
No	8	22	
**NSAIDS**			
Yes	3	13	0.7638
No	38	104	

*LFTs, Liver function tests and NSAIDs, non-steroidal anti-inflammatory drugs.*

### Persistence of LFTs Abnormality During Hospitalization and Risk Factors

Among 117 patients with abnormal LFTs, 52 patients with abnormal LFTs on admission had at least one additional LFTs before discharge. Among them, 23 (44.2%), 35 (67.3%), 7 (13.5%), 4 (7.7%), or 33 (63.5%) patients had elevated ALT, AST, TB, ALP, or GGT, respectively (Table 4). Patients with abnormal LFTs tended to recover from elevated AST (71.4%) or ALP (100%) but not elevated ALT (39.1%), TB (42.9%), and GGT (18.2%). Overall, 38 (73.1%) patients had persistently abnormal LFTs at last assessment. Univariate logistic regression analysis showed that age, hypertension and lymphocyte counts were significantly associated with persistently abnormal LFTs during hospitalization. Among these risk factors, age and lymphocyte counts were independently associated with persistently abnormal LFTs after adjusting use of antibiotics and herbals in multivariate logistic regression (Table 5).

**TABLE 4:** Dynamic changes of LFTs between admission and discharge.

	**Number of patients**	**Number of patients**	**Normalization**
	**with abnormal**	**with normalized**	**rate**
	**LFTs at admission**	**LFTs at discharge**	
ALT	23	9	39.13%
AST	35	25	71.43%
TBIL	7	3	42.86%
ALP	4	4	100%
GGT	33	6	18.18%

*LFTs, Liver function tests; ALT, alanine aminotransferase; AST, aspartate transaminase; ALP, alkaline phosphatase; and GGT, gamma-glutamyltransferase.*

**TABLE 5:** Logistic regression analyses of clinical factors associated with persistently abnormal LFTs during hospitalization.

**Variabe**	**Univariable odds ratio**	***P* value**	**Multivariable odds ratio**	***P* value**
	**(95% CI)**		**(95% CI)**	
Age	0.964 (0.922–1.007)	0.099	0.916 (0.854–0.983)	0.015
Sex, female vs male	0.390 (0.111–1.365)	0.141		
calculated Sp02/FiO2	0.274 (0.222–3.370)	0.312		
Hpertension, yes vs no	4.364 (0.855–22.262)	0.076	6.412 (0.926–44.399)	0.06
Diabetes, yes vs no	1.655 (0.306–8.963)	0.559		
Severity of COVID-19, severe vs non-severe	3.120 (0.605–16.086)	0.174		
Leukocyte, ×10^9^/L	0.959 (0.771–1.193)	0.707		
Neutrophils, ×109/L	1.059 (0.831–1.351)	0.642		
Lymphocyte × 109/L	0.208 (0.635–0.684)	0.010	0.081 (0.012–0.534)	0.009
D-dimer, mg/L	4.299 (0.506–36.520)	0.182		
Albumin, g/L	0.927 (0.818–1.051)	0.239		
Creatinine, μmol/L	1.007 (0.986–1.028)	0.507		
Lactic dehydrogenase, U/L	1.005 (0.999–1.010)	0.105		
C-reactive protein, μg/dL	1.011 (0.998–1.024)	0.106		
PT, s	1.197 (0.883–1.622)	0.247		
Antibiotic administration, yes vs no	1.150 (0.332–3.983)	0.825	0.256 (0.357–1.837)	0.176
Traditional Chinese medicine, yes vs no	0.537 (0.101–2.862)	0.467	0.094 (0.006–1.394)	0.086

*LFTs, Liver function tests and PT, prothrombin time.*

## Discussion

Increasing evidences support that patients with COVID-19 could develop liver injury mainly manifested as abnormal ALT or AST levels accompanied by slightly elevated bilirubin levels (Wang et al., 2020; Chen et al., 2020a; Xu et al., 2020a). Despite the publication of several papers regarding the liver impairment during COVID-19, few has focused on the changes of LFTs and associated risk factors. Consistent with previous studies, our current study demonstrated a high percentage of patients with abnormal LFTs and these patients developed more severe COVID-19 than those without abnormal LFTs. The values of LFTs reversely correlated with the value of calculated PaO2/FiO2, suggesting a linkage between progression of COVID-19 and impairment of liver function. Unexpectedly, a substantial rate of patients discharged with alleviated respiratory symptoms and negative SARS-COV-2 but with abnormal LFTs, suggesting an asynchronous recovery of lung and liver dysfunction in patients with COVID-19. Patients at younger age, with hypertension or low lymphocyte counts were associated with persistent abnormal LFTs during hospitalization in multivariate regression analyses.

The exact pathogenesis of liver injury in SARS-COV-2 infection remains unclear. Possible mechanism includes direct virus-induced cytopathic effects, exacerbation of preexisting liver disease, hypoxemia, drug induced, and overshooting inflammatory responses. Though patients with diagnosed chronic liver disease have been ruled out in this study, there could be a few patients with underlying liver disease that were not captured due to limited access to more advanced imaging technique, such as MRI during the pandemic. Same as SARS-CoV, SARS-CoV-2 uses ACE2 as its entry receptor (Bourgonje et al., 2020). ACE2 expression has been found in bile duct cell, which are crucial in liver regeneration and immune response (Banales et al., 2019). The compensatory regeneration of parenchymal cells from bile duct cells may upregulate ACE2 expression in liver cells that lead to liver impairment. In the current study, AST was most commonly elevated followed by GGT, ALT and ALP. AST is normally present in liver and cardiac cells and has been associated with disease severity (Lei et al., 2020; Qin et al., 2020; Chen et al., 2020b). Previous studies also revealed that AST was more frequently increased than ALT in severe patients upon admission (Guan et al., 2020; Wang et al., 2020). GGT and ALP were both considered indexes of cholangiocytes injury and ALP is more sensitive than GGT for detecting bile duct injury. Our results showed a slight elevation of variables reflecting bile duct injury, suggesting that the duct epithelium injury by SARS-CoV-2 itself is very limited.

Patients with abnormal LFTs were more likely to be male, with poorer oxygenation, lower lymphocyte count, higher neutrophil count, LDH, and CRP comparing to patients with normal LFTs. It was reported that male patients were susceptible to SARS-COV-2 infection and progression of COVID-19 (Li et al., 2020; Zheng et al., 2020) and were therefore at higher risk of having liver impairment. Elevated LDH and CRP, as well as decreased oxygenation index and lymphocyte count were reported to be associated with severe disease progression in patients with COVID-19 (Wynants et al., 2020). The elevated level of LDH in COVID-19 patients is expected because ACE2 is highly expressed in cardiac blood vessels. CRP is a well-known biochemical marker of acute inflammation and is produced primarily in the liver (Ballantyne and Nambi, 2005). Neutrophils elevation manifests as common immune response to infection. The proportion of liver test abnormality in severe patients was significantly higher than in non-severe patients (*p* = 0.018) in our study, which was similar with previous studies (Cai et al., 2020; Huang et al., 2020; Zhang et al., 2020). This could be explained that the inflammatory cytokine storm observed in severe COVID-19 cases may result in multi organ dysfunction, including the liver. Moreover, liver damage may be associated with organ-specific immune response to SARS-CoV-2 or secondary to hypoxemia and systemic inflammation response. Hypoxic hepatitis, also called ischemic hepatitis, is frequently encountered in critically ill patients because of passive congestion or diminished perfusion of the liver. It usually rapidly progresses with a severe elevation of transaminase levels (20 ULN) accompanied by LDH level elevation (Yang et al., 2020). Though our data did not conform with the typical biochemical behavior of hypoxic hepatitis, we found that all liver function indexes, including ALT, AST, TB, ALP, and GGT, were significantly correlated with the oxygenation index. Therefore, liver damage in COVID-19 patients could be related to hypoxia and/or ischemia of hepatocytes, while the specific mechanism requires further study.

Postmortem and liver biopsies of SARS-associated coronavirus showed ballooning of hepatocytes and mild to moderate lobular lymphocytic infiltration, indicating the injury could be caused by either SARS-CoV-2 infection or drug-induced liver injury (Chau et al., 2004; Xu et al., 2020b). Medications of COVID-19 patients in the current study before and after admission included antiviral agents, antibiotics, herbal, NSAIDs and supportive therapies. Antiviral agents included interferon, lopinavir/ritonavir, arbidol, oseltamivir, darunavir, and ribavirin. Antibiotics mainly included quinolone, β-lactam, carbapenem, and oxazolidinone. Herbal medicine mainly refers to Lianhuaqingwen capsule, a manufactured product of the traditional Chinese medicine formula, reported as to ameliorate clinical symptoms of COVID-19 without known side effects on liver function (Hu et al., 2020). 88.6% of patients used lopinavir/ritonavir, a potential therapy for COVID-19 that is also associated with liver injury (Cai et al., 2020; Fan et al., 2020). However, the rate of patients with abnormal LFTs was similar between lopinavir/ritonavir recipients and non-recipients, probably due to the relatively short time of usage or limited sample size. In addition, no significant increase of abnormal LFTs was observed in patients receiving other antiviral agents, antibiotics, herbal or NSAIDs in our study. Therefore, drug-induced liver injury may not be the main reason of liver impairment in COVID-19 patients based on our results.

The high rate of patients discharged with abnormal liver tests in our study could be partly explained by the management strategy of COVID-19 during the pandemic. That is to discharge patients once their SARS-COV-2 being negative to allow more infected patients to be admitted. Therefore, liver dysfunction could be overlooked under such circumstance. Our current data further identified younger age, hypertension and low lymphocyte counts as independent risk factors for persistent liver dysfunction during hospitalization. Whether these patients could benefit from more active treatment on liver dysfunction remains to be investigated. Further studies with longer follow-up are required to investigate the long term outcome of liver function.

The current study has some limitations. It is a retrospective study, which only demonstrates association, not causation. Besides, we are unable to assess the association of liver function abnormalities and mortality, as only 5 patients died in our cohort. Moreover, the duration of abnormal LFTs persistence after COVID-19 recovery remains unknown in our study due to lack of long term follow up. Future study with larger population and longer follow-up period are needed to investigate the mechanism and outcome of liver function abnormalities.

## Conclusion

In this bi-center retrospective study, LFTs abnormality was common in COVID-19 patients and was more prevalent in severe cases than in non-severe cases. A high percentage of patients recovered from COVID-19 but discharged with abnormal LFTs. Younger age, hypertension and low lymphocyte counts were identified as independent risk factors for persistent liver dysfunction during hospitalization. Long-term outcomes of these patients who discharged with abnormal LFTs are needed in follow-up studies.

## Data Availability Statement

The original contributions presented in the study are included in the article/supplementary material, further inquiries can be directed to the corresponding author/s.

## Ethics Statement

The studies involving human participants were reviewed and approved by the Ethics Committee of Bengbu Infectious Disease Hospital and the First Affiliated Hospital of Bengbu Medical College. Written informed consent for participation was not required for this study in accordance with the national legislation and the institutional requirements.

## Author Contributions

QX, HL, WL, and YJ: study concept and design. BG, MS, PW, YS, LH, and TH: data collection. QG and YuZ: data analysis and visualization. QG, ZC, and QX: data interpretation. QG: manuscript drafting. QG, ZC, and QX: critical revision. All authors have approved the final draft submitted.

## Conflict of Interest

The authors declare that the research was conducted in the absence of any commercial or financial relationships that could be construed as a potential conflict of interest.
